# The Prevalence and Epidemiological Features of Ischaemic Heart Disease in Sri Lanka

**DOI:** 10.5334/gh.1330

**Published:** 2024-06-03

**Authors:** Nilmini Wijemunige, Ravindra P. Rannan-Eliya, H. M. M. Herath, Owen O’Donnell

**Affiliations:** 1 Institute for Health Policy, 72 Park Street, Colombo 00200, Sri Lanka; 2 Erasmus School of Economics and Erasmus School of Health Policy & Management, Erasmus University Rotterdam, Postbus 1738, 3000 DR Rotterdam, The Netherlands; 3 Department of Medicine, Faculty of Medicine, University of Ruhuna, Karapitiya, Galle, Sri Lanka

**Keywords:** ischaemic heart disease, cardiovascular disease, angina, Sri Lanka

## Abstract

**Background:**

There is limited evidence on the prevalence of ischaemic heart disease (IHD) and its association with risk factors and socioeconomic status (SES) in low- and middle-income countries (LMICs). Given the relatively high levels of access to healthcare in Sri Lanka, the association of IHD with SES may be different from that observed in other LMICs.

**Objectives:**

To estimate the prevalence of IHD in Sri Lanka, determine its associated risk factors and its association with SES.

**Methods:**

We analysed data from 6,513 adults aged ≥18 years examined in the 2018/19 Sri Lanka Health and Ageing Study. We used the Rose angina questionnaire to classify participants as having angina (Angina+) and used self-report or medical records to identify participants with a history of IHD (History+). The association of Angina+ and History+ with age, ethnicity, sector of residence, education level, household SES wealth quintile, area SES wealth quintile, hypertension, diabetes, smoking, total cholesterol, cholesterol-to-HDL ratio, waist-to-hip ratio and body mass index were analysed in unadjusted and adjusted models. Additional analyses were performed to investigate sensitivity to correction for missing data and to benchmark estimates against evidence from other studies.

**Conclusions:**

We estimated prevalence of History+ of 3.9% (95% CI 3.3%–4.4%) and Angina+ of 3.0% (95% CI 2.4%–3.5%) in adults aged 18 years and over. The prevalence of Angina+ was higher in women than men (3.9% vs. 1.9%, p < 0.001) whilst prevalence of History+ was lower (3.8% vs. 4.0%, p = 0.8), which may suggest a higher rate of undiagnosed IHD in women. A history of IHD was strongly associated with age, hypertension and diabetes status even after adjusting for sociodemographic factors. Though the prevalence of History+ was higher in the most developed area SES tertile and urban areas, History+ was also associated with less education but not household SES, consistent with patterns emerging from other LMICs.

## Introduction

Low- and -middle income countries (LMICs) are experiencing an increasing burden of cardiovascular disease (CVD) as they move through the epidemiological and demographic transitions [1,2]. In high-income countries (HICs), CVD prevalence first increased in more affluent groups before the burden shifted down to groups with lower socioeconomic status (SES). In LMICs, as the burden of CVD increases, it may shift even more rapidly to people of lower SES [1,3].

Sri Lanka is advanced in its epidemiological transition. The proportion of total disability adjusted life years (DALYs) attributable to maternal and child health (MCH) conditions is one-third of the average in LMICs [4], whilst ischaemic heart disease (IHD) (8.5%), stroke (5.6%) and diabetes (8.6%) account for relatively more DALYs. Sri Lankans have access to free universal healthcare and cheap medicines [5]. Process quality of care is high for indicators that need low- to moderate-resources [6]. However, it is not known whether this translates to a different epidemiological pattern of IHD prevalence and its associations with risk factors and with SES.

Like many LMICs, Sri Lanka lacks reliable estimates of IHD prevalence. Using models, the Global Burden of Disease (GBD) project estimates that IHD prevalence in Sri Lanka is 2.2% (males 2.7%, females 1.7%) [4]. Both the crude (2.2%) and age-standardized (2.0%) GBD estimates of IHD prevalence in Sri Lanka are below the average for the South Asia World Bank region (2.6% and 3.6%) and all other World Bank regions, except for Latin America and Caribbean (1.9% and 1.9%), and sub-Saharan Africa (0.1% and 2.1%).

The available local survey-based estimates cover a variety of age groups and populations and are almost more than two decades old. A study of 975 middle-aged males (35–59 years) in the Central Province in 1994 found that 5.4% of participants satisfied the criteria for angina or possible myocardial infarction using the Rose angina questionnaire (RAQ), and a further 3.2% of people satisfied ECG criteria of IHD [7]. In a study of 4,484 people in 7 of the 9 provinces in Sri Lanka in 2005, the estimated age-sex standardized IHD prevalence in Sri Lanka was 9.3% based on RAQ, ECG criteria and treatment for IHD [8]. The prevalence was higher in women (11.3%) than men (7.2%). Another study of 30–65-year-olds in four provinces in Sri Lanka in 2003 found that 4.9% of women and 4.5% of men had angina using RAQ [9,10].

Estimates of IHD prevalence for HICs are typically based on patient databases [11] and self-reported history of IHD or responses on RAQ in population-based surveys [12,13,14]. Most LMICs lack comprehensive patient databases or registries, and few have nationally representative surveys that collect data that can be used to estimate IHD prevalence. The aim of this paper was to estimate the prevalence of IHD, and its association with risk factors and SES, in Sri Lanka, using nationally representative survey data.

## Materials and Methods

### Sample design and selection

We used data from the first wave of the SLHAS, a nationally representative survey of adults aged 18 years and over, conducted from November 2018 to November 2019. Stratified, multi-stage cluster sampling randomly selected one adult from randomly sampled households from 297 primary sampling units defined by Grama Niladhari Divisions (GND) (the smallest administrative unit) located in all 25 districts of Sri Lanka [15]. Interviews were conducted at field clinics. Participants were asked about a history of IHD, hypertension, and diabetes. They were asked to bring their medical records with them to the field clinic. If available, these were also checked for a history of IHD, hypertension or diabetes. Data on age, gender, education level, household assets, housing materials, and water and sanitation facilities were collected through self-reports. Medication history from the previous two weeks was recorded. Weight, height, waist and hip circumference were measured, and body mass index and waist-to-hip ratio was calculated. Participants were instructed to fast. Fasting blood samples were taken by nurses and were tested for blood glucose and lipid profile. People who were fasting and did not report diabetes had oral glucose tolerance tests, and those who had not fasted had random blood glucose and non-fasting lipid profiles.

### Outcomes

The London School of Hygiene Chest Pain Questionnaire—the RAQ—was used to identify people with Rose angina [16,17]. The RAQ has previously been validated for use in Sri Lanka [7]. A person satisfied the criteria for Rose angina (Angina+) if they reported ever having chest pain which appeared upon exertion, was situated at any level of the sternum or left anterolateral chest and arm, which caused the respondent to slow down or stop, and was relieved within ten minutes of rest. A person satisfied the criteria for Rose plus possible infarction if they satisfied the criteria for Rose+ or reported ever having severe chest pain across the front of the chest for thirty minutes or more (Infarction+). We used this outcome in a supplementary analysis.

Participants were defined as having a history of IHD (History+) if either they self-reported when questioned that a doctor had ever told them that they have IHD or they had experienced a myocardial infarction, or their medical records, if brought to the interview, showed a history of IHD. Of the participants with a history of IHD, 88.5% both self-reported and had medical records of this condition, 8.1% self-reported but did not have medical records confirming this and 3.4% had medical records but did not self-report.

We analysed two main outcomes: a) Rose angina (Angina+), and b) history of IHD (History+).

### Risk factors and covariates

Education level was categorized into four groups: no formal education; primary education which included grades 1 to 5; secondary education which included grades 6 to 12, or O-level or A-level certification; and tertiary education which included undergraduate degrees or post-graduate diplomas and degrees. For comparisons with another study, education was also categorized into low (no formal education or primary education), intermediate (secondary education) or high education (tertiary education).

We created a proxy for household SES through a wealth index equal to the first principal component from analysis of household reported durable assets, housing quality, water and sanitation facilities, and other assets (Supplementary File 1, Supplementary Text 1) [18]. Similarly, we calculated area SES from the first principal component of social and economic indicators for each GND obtained from the 2012 census [19]. We created household SES groups from the tertiles and quintiles of the household wealth index and area SES tertile groups from tertiles of the wealth index by GND.

A participant was classified as hypertensive if they a) reported that a doctor had ever told them that they have hypertension or high blood pressure, or b) they were currently taking antihypertensives based on self-report, medical records or medications they brought to the interview or c) brought their medical records to the interview and these stated a history of hypertension, or d) the mean of two blood pressure measurements, taken 10 minutes apart, was 140/90 mmHg or greater [15]. A participant was classified as diabetic if they a) reported that a doctor had ever told them that they have diabetes of high blood sugar, or b) they were currently taking oral or injectable hypoglycaemics based on self-report, medical records or medications they brought to the interview, or c) brought their medical records to the interview and these stated a history of diabetes, or d) gave a blood sample that showed fasting blood glucose ≥ 126 mg/dL, a random glucose ≥ 200 mg/dL, or an oral glucose tolerance test result ≥ 200 mg/dL [19]. A participant who had ever smoked 100 cigarettes or other tobacco products was classified as having a history of smoking.

A participant was recorded as taking statins based on self-report, medical records or medications they brought to the interview belonging to WHO Anatomical Therapeutic Classification (ATC) class C10 (lipid modifying agents).

### Statistical analysis

We estimated IHD prevalence from the sample means of Angina+ and History+. We examined prevalence by gender, age groups, ethnicity, sector of residence (rural, urban, estate, rural/estate), education level, household SES quintile group, and area SES tertile group. We examined variation in prevalence by estimating unadjusted and adjusted odds ratios of each outcome using univariate and multivariate logit models respectively. In the univariate analysis we used the same variables, with age as a continuous variable, and included hypertension status, diabetes status, smoking status, total cholesterol, cholesterol-to-HDL ratio, BMI and waist-to-hip ratio. In the multivariate analysis, we used the same variables as the univariate analysis, except due to similarities in cholesterol and cholesterol-to-HDL ratio, and BMI and waist-to-hip ratio, we ran one model which included total cholesterol and BMI, and a second model which included cholesterol-to-HDL and waist-to-hip ratios. Continuous variables—age, total cholesterol, cholesterol-to-HDL ratio, BMI and waist-to-hip ratio—were standardized to show the odds ratio of one standard deviation change in that variable. A subanalysis was performed to estimate associations between History+ and cholesterol, including statin use and statin intensity in the multivariate regression model using total cholesterol and BMI.

In all analyses, the data were weighted to make them representative of the national population. The original survey design weights were modified using iterative proportional fitting (IPF) to match the district, provincial and national structure along the dimensions of age, sex, sector, and ethnicity [15,19]. When estimating differences in IHD status by diabetes status, the sample weights were further modified to account for possible nonrandom participation in the oral glucose tolerance and fasting blood glucose tests. We multiplied each participant’s original weight by their propensity to provide a glucose test and recalibrated the weights to match the age–sex–ethnicity total weights [19].

We tested the significance of odds ratios in unadjusted and adjusted logit models, using a Wald test for both joint significance of categorical variables (that is, testing whether all levels of a categorical variable have an odds ratio (OR) of one) and specific pairwise comparisons within a categorical variable (that is, testing if the OR between two levels of a categorical variable are the same), and a t-test for continuous variables. All models were adjusted for the complex survey design, accounting for clustering with a finite population correction. All analyses were performed using Stata 17.0 [20].

### Sensitivity analysis

Missing data on Rose angina status and risk factors and covariates potentially make the complete case sample used for the analysis unrepresentative of the population, even after the application of weights. In a sensitivity analysis, we used multiple imputation to impute missing values for Angina status, education category, diabetes status, smoking status, total cholesterol and BMI. We repeated estimation of the univariate and multivariate logit regression models for each outcome using the resulting sample with imputation.

To make comparisons with estimates of the prevalence of myocardial infarction among men aged 35–59 years from another study by Mendis et al. [7], we conducted an additional analysis with the sample restricted to that demographic group and using the Infarction+ outcome.

## Results

We excluded three participants who were less than 18 years old, and 152 participants with missing data for history of IHD, leaving 6,513 (97.7%) participants for analysis. Of these, 6,459 (99.2%) had complete data on RAQ.

Table 1 describes the characteristics of the samples used for Angina+ prevalence and History+ prevalence with data on 6,459 and 6,513 participants respectively. The distribution of demographic and risk factors are very similar between Angina and History samples in both the unweighted and weighted samples. With weighting, the mean age of the History sample was 43.9 years (standard deviation 16.7 years), 23% were diabetic, 27% were hypertensive and 21% had a history of smoking.

**Table 1:** Sociodemographic and risk factor distribution of participants in the Angina sample and History sample.


	ANGINA			HISTORY		
	
	UNWEIGHTED *N*	UNWEIGHTED %/MEAN (SD)	WEIGHTED %/MEAN (SD)	UNWEIGHTED *N*	UNWEIGHTED %/MEAN (SD)	WEIGHTED %/MEAN (SD)

Age	6,459	50.0 (17.2)	43.8 (16.7)	6,513	50.1 (17.2)	43.9 (16.7)

Sex						

Male	3,166	49.0	47.6	3,188	48.9	47.6

Female	3,293	51.0	52.4	3,325	51.1	52.4

Ethnic group						

Sinhala	4,552	70.5	74.9	4,594	70.5	74.9

SL Tamil	1,266	19.6	12.5	1,273	19.5	12.5

Indian Tamil	203	3.1	2.8	205	3.1	2.8

Muslim	412	6.4	9.5	415	6.4	9.5

Other	26	0.4	0.3	26	0.4	0.3

Sector						

Rural	3,566	55.2	70.6	3,590	55.1	70.6

Urban	1,939	30.0	19.6	1,960	30.1	19.6

Estate	166	2.6	0.6	168	2.6	0.7

Rural/Estate	788	12.2	9.2	795	12.2	9.2

Education						

No formal schooling	245	3.8	2.8	252	3.9	2.8

Primary educated	903	14.0	9.9	914	14.0	10.0

Secondary educated	5,041	78.1	82.8	5,074	78.0	82.7

Tertiary educated	263	4.1	4.5	266	4.1	4.5

Household SES quintile						

Poorest	1,535	23.8	19.6	1,547	23.8	19.6

Poorer	1,283	19.9	19.9	1,298	19.9	19.9

Middle	1,194	18.5	19.7	1,200	18.4	19.6

Richer	1,167	18.1	20.0	1,179	18.1	20.0

Richest	1,280	19.8	20.8	1,289	19.8	20.8

Area SES tertile						

Least developed	2,349	36.4	33.2	2,367	36.3	33.2

Middle	1,851	28.7	33.6	1,862	28.6	33.6

Most developed	2,259	35.0	33.2	2,284	35.1	33.3

Hypertension status						

No	4,216	65.3	73.0	4,246	65.2	72.9

Yes	2,243	34.7	27.0	2,267	34.8	27.1

Diabetes status						

No	3,206	67.8	77.1	3,226	67.7	77.1

Yes	1,524	32.2	22.9	1,538	32.3	22.9

Smoking status						

Non-smoker	4,872	77.2	79.2	4,911	77.2	79.2

Ex- or current smoker	1,439	22.8	20.8	1,450	22.8	20.8

Total cholesterol (mean)	6,386	206.2 (47.5)	208.6 (46.9)	6,440	206.1 (47.5)	208.5 (46.9)

Cholesterol-to-HDL ratio (mean)	6,384	4.3 (1.3)	4.4 (1.3)	6,438	4.3 (1.3)	4.4 (1.3)

BMI	6,412	23.8 (4.6)	23.9 (4.6)	6,465	23.8 (4.6)	23.9 (4.6)

Waist-to-hip ratio (mean)	6,429	0.9 (0.1)	0.9 (0.1)	6,483	0.9 (0.1)	0.9 (0.1)


The estimated prevalence of Angina+ was 3.0% (95% CI 2.4%–3.5%) and the prevalence of History+ was 3.9% (95% CI 3.3%–4.4%) in adults aged 18 years and over (Table 2). The prevalence of Angina+ was higher in women than men (3.9% vs. 1.9%, p < 0.001) but was similar to men for History+ (3.8% vs 4.0%, p = 0.8). The prevalence of Angina+ was higher in the poorest household SES quintile (4.4% vs 2.0%, p = 0.04), but the prevalence of History+ was similar (3.8% vs. 4.0%, p = 0.9). The prevalence of History+ was higher in the urban sector than rural sector (6.1% vs. 3.3%, p < 0.001), and in the most developed area SES tertile than least developed (5.1% vs. 2.8%, p < 0.001).

**Table 2:** Prevalence of Angina+ and History+ by sociodemographic category.


	ANGINA+, % (95% CI)				HISTORY+, % (95% CI)			
	
	MALE	FEMALE	ALL		MALE	FEMALE	ALL	
	
	(n = 3,166)	(n = 3,293)	(n = 6,459)		(n = 3,188)	(n = 3,325)	(n = 6,513)	

All	1.9 (1.4–2.5)	3.9 (3.0–4.8)	3.0 (2.4–3.5)	***	4.0 (3.2–4.8)	3.8 (2.9–4.6)	3.9 (3.3–4.4)	

Age category								

<35	1.2 (0.1–2.2)	1.9 (0.7–3.1)	1.5 (0.7–2.3)		0.5 (0.1–0.9)	0.2 †	0.4 (0.1–0.6)	

35–44	1.3 (0.2–2.3)	3.7 (1.9–5.6)	2.5 (1.4–3.7)	*	1.5 (0.4–2.7)	1.3 (0.2–2.4)	1.4 (0.5–2.4)	

45–54	2.1 (0.4–3.8)	4.7 (2.2–7.2)	3.5 (2.0–4.9)		3.1 (1.3–4.8)	2.6 (1.0–4.2)	2.8 (1.8–3.8)	

55–64	2.8 (0.9–4.7)	6.0 (3.6–8.4)	4.5 (3.0–5.9)	*	7.8 (4.9–10.7)	8.4 (5.4–11.4)	8.1 (6.2–10.1)	

65–74	3.5 (1.4–5.5)	6.7 (4.1–9.3)	5.3 (3.7–7.0)	*	14.5 (10.4–18.7)	11.3 (8.0–14.6)	12.6 (10.1–15.2)	

75–84	5.4 (1.9–9.0)	2.2 †	3.7 (1.5–5.8)		15.7 (9.7–21.7)	13.8 (6.0–21.6)	14.7 (10.5–18.8)	

85+	3.4 (1.0–5.8)	5.0 †	4.5 †		12.1 †	4.9 (0.4–9.4)	7.2 (1.3–13.1)	

Ethnicity								

Sinhala	2.1 (1.4–2.9)	4.2 (3.1–5.3)	3.2 (2.5–3.9)	***	4.2 (3.2–5.2)	3.6 (2.7–4.5)	3.9 (3.3–4.5)	

Sri Lankan Tamil	1.6 (0.8–2.4)	1.1 (0.6–1.7)	1.3 (0.8–1.9)		3.8 (2.1–5.6)	2.2 (1.0–3.5)	3.0 (1.9–4.1)	

Indian Tamil	2.9 †	11.6 (1.7–21.5)	7.2 (2.2–12.2)		0.8 †	6.1 †	3.4 †	

Muslim	0.7 (0.1–1.2)	2.8 (0.2–5.5)	1.8 (0.3–3.3)		3.5 †	6.5 (1.5–11.5)	5.1 (2.4–7.8)	

Other	0.0 (0.0–0.0)	13.2 (9.8–16.6)	5.1 †	**	0.0 (0.0–0.0)	13.2 (9.8–16.6)	5.1 †	**

Sector								

Rural	2.0 (1.3–2.7)	3.9 (2.9–5.0)	3.0 (2.3–3.7)	**	3.5 (2.5–4.5)	3.2 (2.3–4.1)	3.3 (2.7–4.0)	

Urban	1.4 (0.5–2.3)	3.3 (1.6–4.9)	2.4 (1.4–3.4)		5.8 (4.0–7.5)	6.3 (3.7–9.0)	6.1 (4.6–7.5)	

Estate	4.3 †	7.9 (2.7–13.2)	5.8 (2.4–9.1)	***	1.9 †	5.6 †	3.4 (1.3–5.5)	

Rural/Estate	2.3 †	4.8 (0.9–8.6)	3.4 (1.6–5.2)		3.9 (1.9–5.9)	2.8 †	3.4 (2.0–4.8)	

Education								

No formal schooling	4.5 (2.7–6.2)	4.7 (2.7–6.6)	4.6 (1.3–7.9)		3.1 †	7.8 (0.7–15.0)	6.3 (2.5–10.1)	

Primary educated	4.6 (1.7–7.5)	7.9 (4.8–10.9)	6.4 (4.6–8.1)		6.4 (3.9–8.9)	9.6 (5.7–13.6)	8.1 (5.7–10.6)	

Secondary educated	1.5 (0.9–2.1)	3.4 (2.4–4.3)	2.5 (1.9–3.1)	***	3.9 (3.0–4.8)	3.0 (2.3–3.8)	3.4 (3.0–3.9)	

Tertiary educated	3.4 (0.2–6.7)	3.1 †	3.3 (0.6–6.0)		1.3 †	0.8 †	1.0 (0.1–2.0)	

Household SES quintile								

Poorest	2.8 (1.0–4.7)	5.4 (3.0–7.7)	4.4 (2.7–6.0)		4.8 (2.9–6.8)	3.2 (1.2–5.1)	3.8 (2.6–5.1)	

Poorer	2.1 (0.8–3.3)	4.1 (2.0–6.2)	3.2 (2.0–4.5)		2.6 (1.4–3.8)	6.0 (3.3–8.7)	4.5 (3.0–6.1)	*

Middle	1.9 (0.6–3.2)	2.6 (0.9–4.3)	2.3 (1.0–3.5)		3.4 (1.4–5.4)	3.0 (1.3–4.6)	3.2 (1.9–4.4)	

Richer	1.9 (0.5–3.2)	4.2 (1.6–6.8)	2.9 (1.7–4.2)		4.0 (2.3–5.6)	3.8 (2.3–5.3)	3.9 (2.9–4.8)	

Richest	1.3 (0.5–2.1)	2.9 (0.8–5.0)	2.0 (1.0–3.1)		4.9 (2.9–7.0)	2.9 (1.1–4.7)	4.0 (2.8–5.2)	

Area SES tertile								

Least developed	2.8 (1.6–4.0)	3.9 (2.8–5.1)	3.4 (2.6–4.2)		2.3 (1.5–3.2)	3.2 (2.1–4.3)	2.8 (1.9–3.7)	

Middle	1.4 (0.6–2.2)	4.4 (2.0–6.7)	3.0 (1.8–4.1)	**	4.2 (2.4–6.0)	3.4 (1.9–4.9)	3.8 (2.8–4.8)	

Most developed	1.6 (0.8–2.3)	3.4 (1.7–5.0)	2.5 (1.5–3.5)	*	5.5 (3.6–7.4)	4.8 (3.0–6.5)	5.1 (4.1–6.1)	


*Notes:* Data are weighted. Significance levels shown for difference between males and females (***p ≤ 0.001, **0.001 < p ≤ 0.01, *0.01 < p ≤ 0.05). *CI* Confidence Interval. †Confidence intervals not shown as lower bounds of CIs were below zero.

Table 3 shows unadjusted and adjusted odds ratios of Angina+ and History+ for each risk factor and covariate. There were significant associations between Angina+ status and age, gender, ethnicity, education level, hypertension, diabetes, and cholesterol-to-HDL ratio, while there were significant associations with History+ status and age, sector of residence, education level, area SES, hypertension, diabetes, total cholesterol, cholesterol-to-HDL ratio and waist-to-hip ratio in unadjusted models.

**Table 3:** Unadjusted and adjusted odds ratios for risk factors of Angina+ and History+ cases.


	ANGINA+		HISTORY+	
	
	UNADJUSTED ODDS RATIO (95% CI)	ADJUSTED ODDS RATIO (95% CI)	UNADJUSTED ODDS RATIO (95% CI)	ADJUSTED ODDS RATIO (95% CI)

Age (years)	1.49 (1.28–1.74) ***	1.28 (0.97–1.68)	3.02 (2.60–3.51) ***	2.46 (1.92–3.15) ***

Gender				

Male	(Ref) ***	(Ref) ***	(Ref)	(Ref)

Female	2.05 (1.4 –2.90)	3.07 (1.64–5.75)	0.95 (0.69–1.32)	0.89 (0.59–1.33)

Ethnicity				

Sinhala	(Ref) ***	(Ref) **	(Ref)	(Ref)

Sri Lankan Tamil	0.40 (0.25–0.64)	0.34 (0.20–0.60)	0.76 (0.50–1.15)	0.73 (0.43–1.25)

Indian Tamil	2.33 (1.08–5.04)	1.29 (0.40–4.11)	0.86 (0.24–3.08)	0.70 (0.18–2.80)

Muslim	0.55 (0.23–1.34)	0.43 (0.14–1.34)	1.32 (0.73–2.39)	0.95 (0.48–1.89)

Other	1.62 (0.21–12.83)	2.28 (0.37–13.93)	1.33 (0.17–10.62)	0.81 (0.14–4.62)

Sector				

Rural	(Ref)	(Ref)	(Ref) **	(Ref)

Urban	0.79 (0.49–1.29)	1.27 (0.56–2.88)	1.87 (1.35–2.58)	1.43 (0.82–2.48)

Estate	1.97 (1.02–3.80)	2.33 (0.90–6.02)	1.02 (0.52–1.99)	1.28 (0.56–2.92)

Rural/Estate	1.14 (0.63–2.06)	0.86 (0.34–2.13)	1.02 (0.64–1.62)	1.57 (0.80–3.11)

Education level				

No formal education	(Ref) ***	(Ref)	(Ref) ***	(Ref)

Primary education	1.41 (0.68–2.91)	1.37 (0.56–3.37)	1.33 (0.70–2.51)	1.48 (0.58–3.77)

Secondary education	0.53 (0.24–1.18)	0.94 (0.36–2.42)	0.53 (0.28–1.03)	1.17 (0.43–3.18)

Tertiary education	0.71 (0.23–2.19)	1.23 (0.34–4.42)	0.16 (0.06–0.43)	0.32 (0.07–1.37)

Household SES quintile				

Poorest	(Ref)	(Ref)	(Ref)	(Ref)

Poorer	0.73 (0.40–1.34)	1.06 (0.46–2.45)	1.19 (0.71–1.99)	1.41 (0.76–2.64)

Middle	0.51 (0.25–1.05)	0.59 (0.24–1.48)	0.82 (0.50–1.34)	0.99 (0.50–1.95)

Richer	0.66 (0.38–1.16)	1.18 (0.51–2.72)	1.02 (0.67–1.55)	0.89 (0.49–1.63)

Richest	0.45 (0.21–0.97)	0.44 (0.16–1.22)	1.05 (0.65–1.71)	0.94 (0.47–1.90)

Area SES tertile				

Least developed	(Ref)	(Ref)	(Ref) **	(Ref)

Middle	0.87 (0.53–1.43)	0.99 (0.53–1.84)	1.38 (0.89–2.13)	0.99 (0.57–1.70)

Most developed	0.74 (0.46–1.19)	0.71 (0.31–1.62)	1.90 (1.28–2.82)	1.30 (0.67–2.52)

Hypertension status				

No hypertension	(Ref) ***	(Ref)	(Ref) ***	(Ref) *

Hypertensive	2.11 (1.51–2.95)	1.58 (0.95–2.61)	5.90 (4.34–8.04)	1.94 (1.17–3.22)

Diabetes status				

No diabetes	(Ref) *	(Ref)	(Ref) ***	(Ref) ***

Diabetes	1.66 (1.10–2.52)	1.27 (0.81–1.98)	3.82 (2.66–5.47)	2.14 (1.46–3.13)

Smoking status				

Non-smoker	(Ref)	(Ref) *	(Ref)	(Ref)

Ex- or current smoker	0.81 (0.55–1.17)	2.09 (1.04–4.21)	1.32 (0.95–1.84)	1.20 (0.72–2.00)

Total cholesterol	0.89 (0.77–1.03)	0.88 (0.74–1.05)	0.59 (0.48–0.73) ***	0.58 (0.46–0.74) ***

Cholesterol-to-HDL ratio	0.76 (0.63–0.91) **	–	0.66 (0.56–0.78) ***	–

BMI	0.97 (0.82–1.15)	0.92 (0.71–1.18)	1.13 (0.98–1.31)	1.06 (0.89–1.27)

Waist-to-hip ratio	1.17 (0.97–1.41)	–	1.51 (1.29–1.76) ***	–


*Notes:* ***p ≤ 0.001, **0.001 < p ≤ 0.01, *0.01 < p ≤ 0.05. *CI* Confidence Interval. Joint significance shown for categorical variables. Odds ratios for continuous variables age, total cholesterol, cholesterol-to-HDL ratio, BMI and waist-to-hip ratio shown for one standard deviation increase in that variable. Cholesterol-to-HDL ratio and waist-to-hip ratio are dropped from the adjusted model.

In adjusted models, people who were older by one standard deviation of age had higher odds of Angina+ (adjusted OR 1.28, 95% CI 0.97–1.68, p = 0.08) and History+ (adjusted OR 2.46, 95% CI 1.92–3.15, p < 0.001). Females had higher odds than males of Angina+ (adjusted OR 3.07, 95% CI 1.64–5.75, p = 0.001) but not of History+ (adjusted OR 0.89, 95% CI 0.59–1.33, p = 0.6). People of Sri Lankan Tamil ethnicity had lower odds of Angina+ compared to people of Sinhala ethnicity (adjusted OR 0.34, 95% CI 0.20–0.60, joint significance: p = 0.002), though the lower odds were not significant for History+. In adjusted models, education level, household and area SES quintiles were not significant. However, a separate analysis (Supplementary File 1, Supplementary Table 1) found that people with low education (no education or primary education) or intermediate level of education (secondary education) had higher adjusted odds (adjusted OR 4.2, 95% CI 1.2–14.4, p = 0.02; adjusted OR 3.4, 95% CI 1.1–10.8, p = 0.04 respectively) of History+ compared to people with high education (tertiary or above).

Hypertensive people had higher odds than normotensive people of History+ (adjusted OR 1.94, 95% CI 1.17–3.22, p = 0.01) though the association with Angina+ was not significant (adjusted OR 1.58, 95% CI 0.95–2.61, p = 0.08). Previous or current smoking was associated with Angina+ (adjusted OR 2.09, 95% CI 1.04–4.21, p = 0.04), but the association with History+ was not significant (adjusted OR 1.20, 95% CI 0.72–2.00, p = 0.5). A one standard deviation increase in total cholesterol and cholesterol-to-HDL ratio was associated with lower odds of History+ (adjusted OR 0.58, 95% CI 0.46–0.74, p < 0.001; 0.72, 95% CI 0.58–0.90, p = 0.004 respectively) (Supplementary File 1, Supplementary Table 2). However, the association with total cholesterol was weaker after controlling for statin use (adjusted OR 0.79, 95% CI 0.62–1.01, p = 0.06) and intensity of statin use (adjusted OR 0.81, 95% CI 0.64–1.02, p = 0.07) (Supplementary File 1, Supplementary Table 3). BMI and waist-to-hip ratio were not significant in the adjusted models.

Sensitivity analysis with imputed values for angina status and covariates with missing data gave similar results to the complete-cases sample analysis, with a stronger association of hypertension with Angina+ and History+, and education level with Angina+ (Supplementary File 1, Supplementary Table 4).

**Figure 1:**
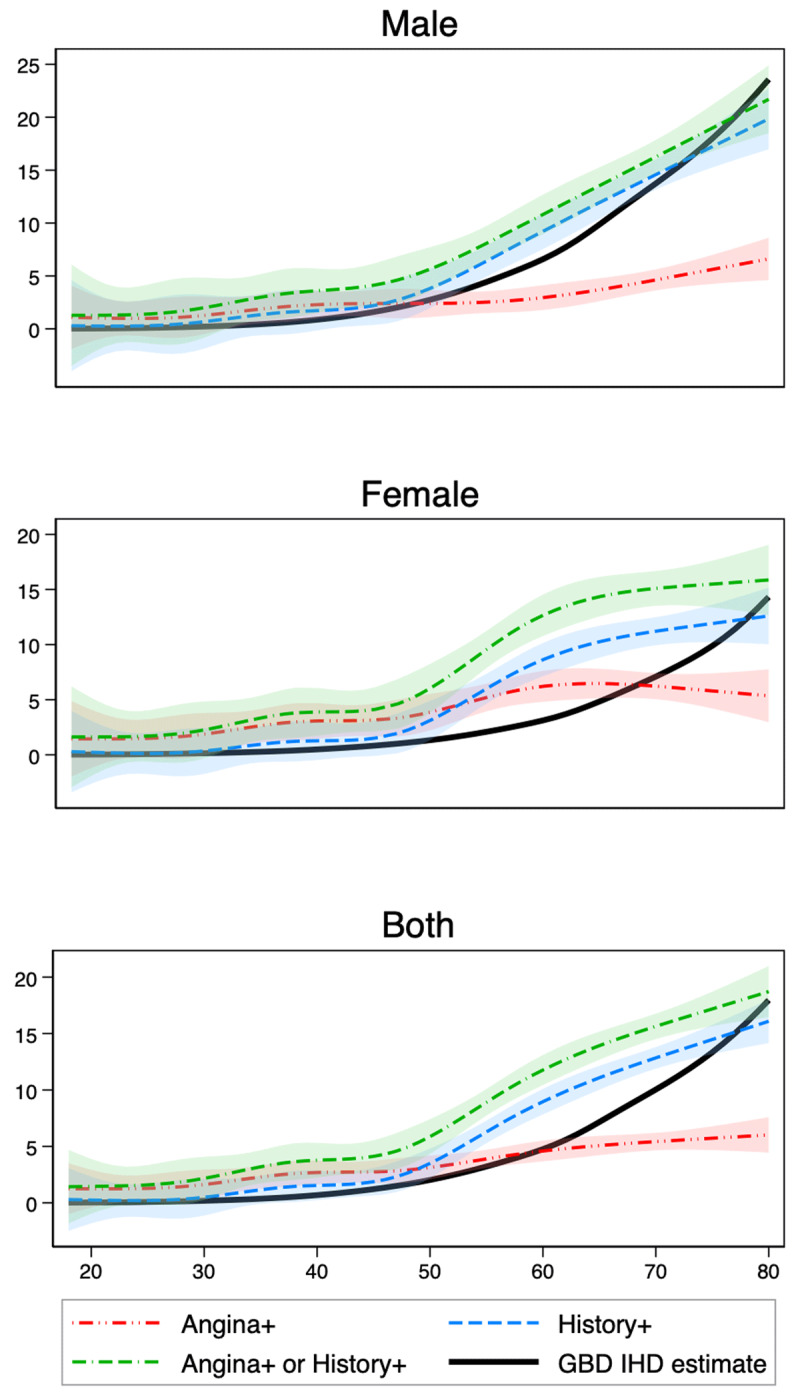
Comparison of smoothed IHD prevalence by age and gender, using Angina+ and History+ criteria with IHD prevalence estimates from Global Burden of Disease. *Notes:* Smoothed prevalence by age are shown for Angina+, History+, and Angina+ or History+, fitting cubic splines with six knots to allow for non-linear relationships using weighted data for participants aged 18–80 years. Shaded regions represent 95% confidence intervals. GBD = Global Burden of Disease study, IHD = Ischaemic Heart Disease.

When restricted to the population aged 40 years and over, the prevalence of Angina+ was 3.7% (95% CI 3.0%–4.5%) and History+ was 6.7% (95% CI 5.7%–7.6%). Assuming no IHD in the population younger than 18 years of age, the prevalence of Angina+ or History+ in the total population is 3.8% (95% CI 3.4%–4.3%) whilst the prevalence of Angina+ is 1.9% (95 CI 1.5%–2.2%) and History+ is 2.5% (95% CI 2.1%–2.8%). Analysis of males aged between 35 years and 59 years, combining both angina and possible myocardial infarction on RAQ gave a prevalence of Infarction+ of 8.9% (95% CI 7.7%–10.2%). Restriction to the population aged 30–65 years gave an estimated prevalence of Angina+ in males as 2.2% (95% CI 1.4%–2.9%) and females as 4.3% (95% CI 3.2%–5.4%). The prevalence of History+ is somewhat higher than the prevalence of IHD estimated by the GBD study when analysed by age and gender, particularly between the ages of 50–70, with the difference more pronounced for women (Figure 1).

## Discussion

Estimating IHD prevalence in LMICs is challenging given the lack of adequate data, especially representative population surveys with measurements that allow identification of IHD. The RAQ has been used in a wide range of population surveys in both HICs and some LMICs to estimate prevalence of angina, which has usually been found to be associated with a higher risk of future coronary artery events [21,22]. Our study presents the first known estimates of IHD prevalence in Sri Lanka using nationally representative data, with analysis of the correlation of IHD with known risk factors and sociodemographic features.

The prevalence of IHD in Sri Lanka using RAQ or IHD history, appears to be high at 3.8%, with the estimated prevalence higher than estimates produced by the GBD study (2.2%). Estimates of IHD for females in this study are higher than GBD estimates, and females have higher – almost double the odds – of being Angina+ than males, confirming a pattern of female preponderance for angina symptoms globally. People in urban areas and the most developed SES tertile had higher odds of History+. People with hypertension and diabetes also had higher odds of History+ even after adjustment.

Compared to data collected from four Sri Lankan provinces two decades ago, which used the Angina+ definition and a sample aged 30–65 years, our study found a lower prevalence for men (2.2% vs. 4.5% of men and 4.3% vs. 4.9% of women) [9,10]. Meanwhile our study prevalence was higher compared to estimates from one province more than three decades ago using the Ischaemia+ definition in people aged 35–59 years (8.9% vs. 5.4%) [7]. The estimated prevalence of angina (3.0%) is within the bounds of angina on RAQ in a metanalysis of 74 studies of 31 LMIC and highincome countries which found the prevalence of angina on RAQ ranged from 0.73% to 14.4% [9].

The overall prevalence of Angina+ or History+, 3.8% (95% CI 3.4%–4.3%), is higher than GBD study estimates for IHD (2.2%, 95% CI 1.9%–2.5%) for Sri Lanka. A prevalence of 3.8% is similar to the crude prevalence of IHD estimated by GBD for the Middle East and North Africa region (3.6%, 95% CI 3.4%–3.9%), which GBD reports as the World Bank region with the second highest prevalence of IHD [4]. The GBD uses similar definitions for IHD prevalence, including angina based on the RAQ and myocardial infarction, performing modelling on data from 61 countries to generate country-specific estimates [23]. Similar to our study, the GBD definition does not include estimates based on electrocardiograph (ECG) evidence for prior MI citing limited specificity and sensitivity. The GBD uses modelling of incident myocardial infarction, and scales angina prevalence based on RAQ, to angina prevalence using claims data from the United States, which may account for the lower prevalence of IHD in the GBD study. Nevertheless, restricting prevalence estimates to History+, the prevalence is still somewhat higher in this study than GBD estimates, particularly for Sri Lankan women aged 50–70 years. Recent findings in diabetes prevalence using the same survey data also found far higher rates in Sri Lanka than what was estimated by the NCD Risk Factor Collaboration (NCD-RisC) [19,24], suggesting that current global estimates for metabolic syndrome-related conditions may be systematically underestimated for Sri Lanka.

Our study found a higher prevalence and odds ratio of Angina+, which focuses on angina symptoms, in women than men. Globally, the prevalence of angina is typically reported to be higher in females than in males, although males were more often diagnosed with IHD in most populations in the world [9]. Research suggests that there could be differences in the symptoms that women with IHD report compared to men, and that they are more likely to have non-obstructive coronary artery disease than obstructive disease, which, amongst other factors, can lead to underdiagnosis of IHD [25,26]. Whilst this study focused on typical and not atypical symptoms, is not clear if even women with typical symptoms of angina are as likely to seek medical care as men, and if they do, whether physicians diagnose them with IHD [9]. Furthermore, women with typical symptoms are less likely than men to have obstructive disease on angiogram [27,28] or can also have normal coronary arteries [29]. However, these women still have higher rates of cardiovascular events than women with no symptoms [28,29,30,31]. Importantly, there is evidence that women with typical symptoms may receive less medical intervention than men [27], possibly because women with symptoms and a normal or non-obstructive angiogram would be likely to receive little medical treatment for IHD [28,29]. Given that our study is in line with global findings that women have a higher prevalence of angina symptoms, but a similar prevalence of diagnosed IHD as men, it is also possible then that women in Sri Lanka with IHD are underdiagnosed, and that these underdiagnosed symptomatic women could have a poorer prognosis than those without symptoms. Therefore, it is important to ensure there is an equal focus on diagnosing IHD in women, particularly those who present with symptoms of angina.

As expected, increasing age, hypertension and diabetes were strongly associated with History+. Furthermore, the prevalence of History+ was higher in people living in the most developed area SES tertile and urban areas. Though the prevalence of Angina+ was higher in the poorest household quintile, a household wealth gradient was not seen for History+. Though statistically significant ORs were not seen for the household SES gradient for Angina+, there may again be a possibility of underdiagnosis of IHD in the poorest household SES quintile as was seen for women compared to men, and this needs further investigation with longitudinal data.

Typically, CVDs in LMICs are thought to shift from a disease concentrated in the affluent, to one concentrated in the poor: a demographic shift that was seen in high income countries. However, the speed of this transition from rich to poor may be faster in LMICs than historically seen elsewhere [32]. In subnational data collected in 2005/6, the prevalence of CVD risk factors – diabetes, obesity and hypertension – was higher in urban areas [33,34,35], which were generally higher income areas [33], with obesity also confirmed to be higher in the rich. The pattern remained largely the same in 2018/9 where the prevalence of diabetes and hypertension was higher in urban areas, the most developed area SES tertile, and richer household quintiles. However, the development of ischaemic heart disease is multifactorial and arises due to a combination of risk factors and medical management of risk factors. Countering the pattern of metabolic conditions concentrating in urban and affluent populations, are CVD risk factors such as smoking which may be higher in the poor [36,37], and hypertension, which also has a high prevalence in rural areas [15]. However, in this study, it appears that IHD, proxied by History+, is still more common in urban and more affluent areas in Sri Lanka.

Similar to a prospective study of CVD conducted in 20 countries, including LMICs, we found that History+ had a stronger association with level of education than household wealth [38]. After adjusting for age, gender, ethnicity, sector, household and area SES, and CVD risk factors, people with low levels of education had higher odds than people with high levels of education of History+, whilst no such pattern was seen for household SES. While the lack of a gradient of History+ for household SES could hold if there is an element of underdiagnosis of IHD in poorer quintiles, it is unlikely that rates of underdiagnosis apply to household SES but not to lower education levels. It is generally considered that Sri Lankans, including the less affluent, have access to universal healthcare with a focus on primary prevention [5,39], and access to cheap medicines [40,41,42,43], all of which may contribute to better primary prevention of IHD and management of conditions which increase the risk of IHD [42]. Therefore, it is important that research in the Sri Lankan context on the development, diagnosis, treatment and control of IHD and its risk factors such as diabetes and hypertension, not only focus on wealth gradients, but level of education as well.

Some known risk factors for IHD did not appear to be significant for either Angina+ or History+ or both. For example, the odds of History+ reduced for one standard deviation increase of total cholesterol (OR 0.59). However, this could be due to statin treatment of History+ participants, which is part of standard treatment guidelines. In multivariate models including statin treatment, and statin treatment intensity, the odds ratio increased to 0.79 and 0.81 and was no longer statistically significant. The association of History+ with past or current smoking was not strong, and could be due to underreporting of smoking in participants. The odds ratios associated with anthropometric measurements are mixed. Higher odds of History+ were associated with a one standard deviation increase in waist-to-hip ratios and with BMI to a lesser extent in the unadjusted model, but neither were significant in the adjusted models. Meanwhile an increase in BMI was associated with higher odds of History+, but less so for Angina+, similar to findings in India [44]. The association of anthropometric measurements with IHD risk in South Asians is not fully understood, and there is debate as to which anthropometric measure is more closely correlated with IHD [45]. A separate analysis using various obesity indices, such as BMI, waist circumference, waist-to-hip circumference, waist circumference-to-height and body fat analysis and their association with IHD and other IHD risk factors would be useful.

Our study may have implications for the CVD risk screening tool which estimates the 10 year risk of developing CVD, produced by the WHO in 2019 [46]. Data from the 2017 GBD study, which uses similar techniques to the 2019 study, was used to recalibrate risk models to age-sex-region specific incidences, to create region-specific CVD risk calculators and charts for use in CVD risk screening programs. Furthermore, the incidence of CVD predicted for the SLHAS cohort using the WHO-2019 risk tool for Sri Lanka closely follows the incidence of IHD estimated by the 2019 GBD study [47]. Given the finding of possible underestimation of IHD prevalence in the GBD study, particularly in women, it is important to monitor and validate the performance of the WHO-2019 risk screening tool using longitudinal data as it becomes available in the future.

There are some limitations in this study. The prevalence based on the respondent’s recall of a doctor diagnosis of IHD or medical records kept by the respondent may be misclassified and possibly underestimates the true prevalence. Prevalence based on RAQ, which is neither specific nor highly sensitive for IHD provides support for the prevalence of IHD. In the absence of registration data to further support these findings, an analysis of the ECG records of study participants using specific criteria for coronary heart disease may provide further insight to the prevalence of IHD.

This is a cross-sectional study, and it did not account for survival bias, or changes in risk factors that may occur with aggressive treatment and behavioural changes following the development of IHD. Future follow-up of participants who have not reported IHD will be useful to check whether they developed IHD, and assess the relationship between baseline CVD risk factors and angina status on RAQ in the Sri Lankan population. A population-based cohort study of a population aged 20–54 years in Norway, for example, suggested that the increased risk of IHD of participants with angina based on a shortened RAQ was explained largely by known cardiovascular disease risk factors [13].

## Conclusions

This study provides the first survey-based national estimates of the prevalence of IHD in Sri Lanka. In a setting without comprehensive registration data of IHD, surveys of self-reported IHD and angina using RAQ can provide credible estimates of prevalence. As expected, people who were older or had hypertension or diabetes had higher odds of having IHD. The strength of the association with age, hypertension, and diabetes in adjusted models and lack of association with wealth quintiles could be consistent with other indicators of equality in access to basic healthcare. Nevertheless, there was an association of IHD with lower education levels, consistent with studies from other LMICs which warrants further attention. The prevalence of angina was higher in women, however self-reported IHD was slightly higher in men, consistent with many international studies, and suggests a possible underdiagnosis of IHD in women. Further studies analysing ECG data to confirm these patterns, follow-up of this current cohort to detect incident IHD, and analysing risk factor distribution amongst various socioeconomic groups will provide a more complete picture of the epidemiology of IHD in Sri Lanka.

## Data Accessibility Statement

Data are available on reasonable request. The study data were provided by the SLHAS Consortium, which has adopted an Open Data policy which will provide access to SLHAS Wave 1 data from 2024, on application to the Consortium by interested researchers.

## Additional File

The additional file for this article can be found as follows:

10.5334/gh.1330.s1 Supplementary File 1. Supplementary Tables 1 to 4 and Supplementary Text 1.

## Abbreviations

ATC: Anatomical therapeutic classification

CI: confidence interval

CVD: cardiovascular disease

DALYs: disability adjusted life years

GBD: Global Burden of Disease

GND: Grama Niladhari Division

HICs: high-income countries

IHD: ischaemic heart disease

LMIC: low- and middle-income countries

OR: odds ratio

RAQ: Rose angina questionnaire

SES: socioeconomic status

SLHAS: Sri Lankan Health and Ageing Study
