# Supplementary material for: The Prevalence and Epidemiological Features of Ischaemic Heart Disease in Sri Lanka
Source: Glob Heart. 2024 Jun 3;19(1):49. doi: 10.5334/gh.1330 (PMC11160409; doi:10.5334/gh.1330)
Supplement: Supplementary File 1. — Supplementary Tables 1 to 4 and Supplementary Text 1. [file gh-19-1-1330-s1.pdf]

SUPPLEMENTARY FILE 1

The prevalence and epidemiological features of ischaemic heart disease in Sri Lanka

Nilmini Wijemunige, Ravindra P. Rannan-Eliya, H.M.M. Herath, Owen O'Donnell

Supplementary Table 1 Unadjusted and adjusted odds ratio for Angina+ and History+ cases using 3-level categorization of education and SES.......... 3

Supplementary Table 2 Adjusted odds ratio of Angina+ and History+ by sociodemographic category, using cholesterol-to-HDL ratio and waist-to-hip ratio.......... 2

Supplementary Table 3 Adjusted odds ratio for History+ cases with and without controlling for statin use and intensity.......... 4

Supplementary Table 4 Unadjusted and adjusted odds ratios for Angina+ and History+ cases using imputed data.......... 5

Supplementary Text 1 Estimation of household socioeconomic status using principal components analysis.......... 6

Supplementary Table 1 Unadjusted and adjusted odds ratio for Angina+ and History+ cases using 3-level categorization of education and SES

	Unadjusted			Adjusted		
	**Odds ratio (95% CI)**	**p-value (Group)**	**p-value (Versus reference)**	**Odds ratio (95% CI)**	**p-value (Group)**	**p-value (Versus reference)**
**Angina+**						
Education level						
High (Ref)		0.000 ***	-		0.4	-
Low	1.9 (0.8 - 4.6)	-	0.2	1.1 (0.4 - 3.5)	-	0.9
Intermediate	0.7 (0.3 - 1.8)	-	0.5	0.8 (0.3 - 2.2)	-	0.6
Household SES Tertile						
High (Ref)		0.06	-		0.3	-
Low	1.9 (1.1 - 3.3)	-	0.03 *	1.7 (0.8 - 3.9)	-	0.2
Medium	1.2 (0.7 - 2.0)	-	0.6	1.3 (0.6 - 2.7)	-	0.6
**History+**						
Education level						
High (Ref)		0.000 ***	-		0.1	-
Low	7.9 (3.1 - 20.1)	-	0.000 ***	4.2 (1.2 - 14.4)	-	0.02 *
Intermediate	3.4 (1.3 - 8.4)	-	0.010 **	3.4 (1.1 - 10.8)	-	0.04 *
Household SES Tertile						
High (Ref)		0.91	-		0.8	-
Low	0.9 (0.7 - 1.3)	-	0.7	1.0 (0.6 - 1.7)	-	0.8
Medium	0.9 (0.6 - 1.4)	-	0.7	1.2 (0.7 - 2.2)	-	0.5

Notes: *** $p \leq 0.001$, ** $0.001 < p \leq 0.01$, * $0.01 < p \leq 0.05$. *CI* Confidence Interval. Joint significance shown for p-value (Group). Significance tested against reference group for p-value (Versus reference). Adjusted estimates used multivariate regressions with the same covariates as shown in Table 2, with modifications for education level and household SES tertile as specified in this table.

Supplementary Table 2 Adjusted odds ratio of Angina+ and History+ by sociodemographic category, using cholesterol-to-HDL ratio and waist-to-hip ratio

	Angina+		History+	
	Unadjusted odds ratio (95% CI)	Adjusted odds ratio (95% CI)	Unadjusted odds ratio (95% CI)	Adjusted odds ratio (95% CI)
Age (years)	1.49 (1.28 - 1.74) ***	1.22 (0.94 - 1.58)	3.02 (2.60 - 3.51) ***	2.32 (1.83 - 2.94) ***
Gender				
Male	(Ref) ***	(Ref) ***	(Ref)	(Ref)
Female	2.05 (1.44 - 2.90)	2.84 (1.54 - 5.23)	0.95 (0.69 - 1.32)	0.78 (0.51 - 1.18)
Ethnicity				
Sinhala	(Ref) ***	(Ref) **	(Ref)	(Ref)
Sri Lankan Tamil	0.40 (0.25 - 0.64)	0.34 (0.20 - 0.60)	0.76 (0.50 - 1.15)	0.82 (0.49 - 1.36)
Indian Tamil	2.33 (1.08 - 5.04)	1.38 (0.42 - 4.53)	0.86 (0.24 - 3.08)	0.74 (0.18 - 3.03)
Muslim	0.55 (0.23 - 1.34)	0.42 (0.14 - 1.31)	1.32 (0.73 - 2.39)	0.92 (0.45 - 1.89)
Other	1.62 (0.21 - 12.83)	2.50 (0.44 - 14.14)	1.33 (0.17 - 10.62)	1.05 (0.19 - 5.66)
Sector				
Rural	(Ref)	(Ref)	(Ref) **	(Ref)
Urban	0.79 (0.49 - 1.29)	1.26 (0.56 - 2.86)	1.87 (1.35 - 2.58)	1.50 (0.86 - 2.60)
Estate	1.97 (1.02 - 3.80)	2.22 (0.85 - 5.86)	1.02 (0.52 - 1.99)	1.27 (0.61 - 2.68)
Rural/Estate	1.14 (0.63 - 2.06)	0.83 (0.33 - 2.09)	1.02 (0.64 - 1.62)	1.51 (0.76 - 3.02)
Education level				
No formal education	(Ref) ***	(Ref)	(Ref) ***	(Ref)
Primary education	1.41 (0.68 - 2.91)	1.32 (0.53 - 3.30)	1.33 (0.70 - 2.51)	1.47 (0.59 - 3.63)
Secondary education	0.53 (0.24 - 1.18)	0.86 (0.33 - 2.25)	0.53 (0.28 - 1.03)	1.13 (0.44 - 2.92)
Tertiary education	0.71 (0.23 - 2.19)	1.17 (0.32 - 4.25)	0.16 (0.06 - 0.43)	0.35 (0.09 - 1.39)
Household SES quintile				
Poorest	(Ref)	(Ref)	(Ref)	(Ref)
Poorer	0.73 (0.40 - 1.34)	1.03 (0.44 - 2.38)	1.19 (0.71 - 1.99)	1.33 (0.70 - 2.52)
Middle	0.51 (0.25 - 1.05)	0.57 (0.22 - 1.42)	0.82 (0.50 - 1.34)	0.97 (0.50 - 1.88)
Richer	0.66 (0.38 - 1.16)	1.11 (0.49 - 2.52)	1.02 (0.67 - 1.55)	0.88 (0.47 - 1.64)
Richest	0.45 (0.21 - 0.97)	0.41 (0.15 - 1.17)	1.05 (0.65 - 1.71)	0.91 (0.45 - 1.86)
Area SES tertile				
Least developed	(Ref)	(Ref)	(Ref) **	(Ref)
Middle	0.87 (0.53 - 1.43)	0.98 (0.52 - 1.84)	1.38 (0.89 - 2.13)	1.07 (0.62 - 1.82)
Most developed	0.74 (0.46 - 1.19)	0.73 (0.31 - 1.69)	1.90 (1.28 - 2.82)	1.31 (0.66 - 2.58)
Hypertension status				
No hypertension	(Ref) ***	(Ref)	(Ref) ***	(Ref) **
Hypertensive	2.11 (1.51 - 2.95)	1.50 (0.92 - 2.47)	5.90 (4.34 - 8.04)	2.03 (1.24 - 3.31)
Diabetes status				
No diabetes	(Ref) *	(Ref)	(Ref) ***	(Ref) ***
Diabetes	1.66 (1.10 - 2.52)	1.22 (0.77 - 1.93)	3.82 (2.66 - 5.47)	2.28 (1.56 - 3.35)
Smoking status				
Non-smoker	(Ref)	(Ref) *	(Ref)	(Ref)
Ex- or current smoker	0.81 (0.55 - 1.17)	2.07 (1.02 - 4.20) *	1.32 (0.95 - 1.84)	1.12 (0.69 - 1.84)
Total cholesterol	0.89 (0.77 - 1.03)	-	0.59 (0.48 - 0.73) ***	-
Cholesterol-to-HDL ratio	0.76 (0.63 - 0.91) **	0.78 (0.62 - 0.97) *	0.66 (0.56 - 0.78) ***	0.72 (0.58 - 0.90) **
BMI	0.97 (0.82 - 1.15)	-	1.13 (0.98 - 1.31)	-
Waist-to-hip ratio	1.17 (0.97 - 1.41)	1.11 (0.85 - 1.44)	1.51 (1.29 - 1.76) ***	1.11 (0.93 - 1.34)

Notes: *** $p \leq 0.001$, ** $0.001 < p \leq 0.01$, * $0.01 < p \leq 0.05$. *CI* Confidence Interval. Joint significance shown for categorical variables. Odds ratios for continuous variables age, cholesterol-to-HDL ratio, waist-to-hip ratio, total cholesterol and BMI shown for one standard deviation increase in that variable. Total cholesterol and BMI are dropped from the adjusted model.

Supplementary Table 3 Adjusted odds ratio for History+ cases with and without controlling for statin use and intensity

	History + Adjusted odds ratio (95% CI)		
	**Without controlling for statin use**	**Controlling for statin use**	**Controlling for statin intensity**
Total cholesterol	0.58 (0.46 - 0.74) ***	0.79 (0.62 - 1.01)	0.81 (0.64 - 1.02)
On statin			
No	-	(Ref) ***	-
Yes	-	5.01 (2.67 - 9.40)	-
Statin intensity			
No statin	-	-	(Ref) ***
Low intensity	-	-	2.20 (0.68 - 7.09)
Moderate intensity	-	-	4.56 (2.31 - 9.02)
High intensity	-	-	22.09 (9.70 - 50.30)

Notes: *** $p \leq 0.001$, ** $0.001 < p \leq 0.01$, * $0.01 < p \leq 0.05$. *CI* Confidence Interval. Joint significance shown for categorical variables. Odds ratios for total cholesterol shown for one standard deviation increase in that variable. Multivariate regressions used the same covariates as shown in Table 2, with and without controlling for statin use. Statin intensity obtained from Table 3 in the 2018 American Heart Association [1].

References

1. Grundy SM, Stone NJ, Bailey AL, et al. 2018 AHA/ACC/AACVPR/AAPA/ABC/ ACPM/ADA/AGS/APhA/ASPC/NLA/PCNA Guideline on the Management of Blood Cholesterol: A Report of the American College of Cardiology/American Heart Association Task Force on Clinical Practice Guidelines. Circulation 2019;139(25):e1082-e143. doi: 10.1161/CIR.0000000000000625

Supplementary Table 4 Unadjusted and adjusted odds ratios for Angina+ and History+ cases using imputed data

	Angina+		History+	
	Unadjusted odds ratio (95% CI)	Adjusted odds ratio (95% CI)	Unadjusted odds ratio (95% CI)	Adjusted odds ratio (95% CI)
Age (years)	1.50 (1.29 - 1.76) ***	1.22 (1.00 - 1.48) *	3.02 (2.60 - 3.51) ***	2.29 (1.85 - 2.83) ***
Gender				
Male	(Ref) ***	(Ref) ***	(Ref)	(Ref)
Female	2.06 (1.45 - 2.92)	2.33 (1.43 - 3.80)	0.95 (0.69 - 1.32)	0.92 (0.63 - 1.34)
Ethnicity				
Sinhala	(Ref) ***	(Ref) ***	(Ref)	(Ref)
Sri Lankan Tamil	0.41 (0.26 - 0.64)	0.32 (0.19 - 0.53)	0.76 (0.50 - 1.15)	0.65 (0.40 - 1.08)
Indian Tamil	2.28 (1.05 - 4.94)	1.64 (0.67 - 4.00)	0.86 (0.24 - 3.08)	0.73 (0.22 - 2.39)
Muslim	0.54 (0.23 - 1.31)	0.53 (0.21 - 1.38)	1.32 (0.73 - 2.39)	0.86 (0.51 - 1.45)
Other	1.61 (0.20 - 12.68)	1.12 (0.14 - 8.95)	1.33 (0.17 - 10.62)	0.44 (0.06 - 3.39)
Sector				
Rural	(Ref)	(Ref)	(Ref) **	(Ref)
Urban	0.79 (0.49 - 1.28)	0.98 (0.50 - 1.89)	1.87 (1.35 - 2.58)	1.46 (1.00 - 2.14)
Estate	1.97 (1.03 - 3.78)	2.19 (0.90 - 5.35)	1.02 (0.52 - 1.99)	1.13 (0.51 - 2.52)
Rural/Estate	1.12 (0.62 - 2.04)	1.09 (0.53 - 2.26)	1.02 (0.64 - 1.62)	1.30 (0.73 - 2.30)
Education level				
No formal education	(Ref) ***	(Ref) *	(Ref) ***	(Ref)
Primary education	1.44 (0.69 - 2.98)	1.74 (0.82 - 3.72)	1.33 (0.70 - 2.51)	1.75 (0.85 - 3.60)
Secondary education	0.53 (0.24 - 1.18)	0.99 (0.43 - 2.28)	0.53 (0.28 - 1.03)	1.34 (0.63 - 2.85)
Tertiary education	0.71 (0.23 - 2.18)	1.75 (0.57 - 5.35)	0.16 (0.06 - 0.43)	0.45 (0.13 - 1.53)
Household SES quintile				
Poorest	(Ref)	(Ref)	(Ref)	(Ref)
Poorer	0.75 (0.41 - 1.37)	0.90 (0.47 - 1.73)	1.19 (0.71 - 1.99)	1.33 (0.75 - 2.37)
Middle	0.51 (0.25 - 1.04)	0.65 (0.32 - 1.33)	0.82 (0.50 - 1.34)	0.87 (0.50 - 1.52)
Richer	0.66 (0.38 - 1.16)	0.90 (0.46 - 1.75)	1.02 (0.67 - 1.55)	0.97 (0.57 - 1.64)
Richest	0.45 (0.21 - 0.97)	0.61 (0.25 - 1.44)	1.05 (0.65 - 1.71)	0.99 (0.54 - 1.82)
Area SES tertile				
Least developed	(Ref)	(Ref)	(Ref) **	(Ref)
Middle	0.88 (0.54 - 1.44)	0.88 (0.54 - 1.44)	1.38 (0.89 - 2.13)	0.95 (0.56 - 1.62)
Most developed	0.75 (0.47 - 1.20)	0.81 (0.42 - 1.54)	1.90 (1.28 - 2.82)	1.09 (0.62 - 1.91)
Hypertension status				
No hypertension	(Ref) ***	(Ref) **	(Ref) ***	(Ref) ***
Hypertensive	2.15 (1.54 - 3.00)	1.68 (1.16 - 2.43) **	5.90 (4.34 - 8.04)	2.29 (1.55 - 3.39)
Diabetes status				
No diabetes	(Ref) *	(Ref)	(Ref) ***	(Ref) **
Diabetes	1.53 (1.06 - 2.22)	1.16 (0.76 - 1.78)	4.54 (3.26 - 6.32)	1.82 (1.25 - 2.67)
Smoking status				
Non-smoker	(Ref)	(Ref)	(Ref)	(Ref)
Ex- or current smoker	0.76 (0.53 - 1.11)	1.31 (0.79 - 2.18)	1.34 (0.96 - 1.87)	1.33 (0.92 - 1.91)
Total cholesterol	0.88 (0.76 - 1.02)	0.85 (0.74 - 0.99) *	0.59 (0.48 - 0.73) ***	0.61 (0.49 - 0.76) ***
Cholesterol-to-HDL ratio	0.76 (0.63 - 0.91) **	-	0.66 (0.56 - 0.78) ***	-
BMI	0.98 (0.83 - 1.15)	0.95 (0.79 - 1.13)	1.13 (0.98 - 1.30)	1.09 (0.94 - 1.27)
Waist-to-hip ratio	1.16 (0.97 - 1.40)	-	1.50 (1.29 - 1.75) ***	-

Notes: *** $p \leq 0.001$, ** $0.001 < p \leq 0.01$, * $0.01 < p \leq 0.05$. *CI* Confidence Interval. Joint significance shown for categorical variables. Odds ratios for continuous variables age, cholesterol-to-HDL ratio, waist-to-hip ratio, total cholesterol and BMI shown for one standard deviation increase in that variable. Total cholesterol and BMI are dropped from the adjusted model.

Supplementary Text 1 Estimation of household socioeconomic status using principal components analysis

The SLHAS Wave 1 uses an asset index approach to generate a proxy measure of each household's living standard. The index was computed by using principal components analysis (PCA) of a set of household-level variables relating to asset ownership or household characteristics. Variables were selected from those used in recent Sri Lanka Household Income and Expenditure Surveys conducted by the Department of Census and Statistics, selecting those with most predictive performance, and excluding some assets that are only relevant to agricultural households (e.g., tractor, thresher, fishing equipment). Variables were either dichotomous (e.g., household has a car) or categorical (e.g., type of drinking water source), apart from one ordinal variable (number of bedrooms). Dichotomous variables consisted of whether the household possessed each of the following items: radio/cassette player, television, VCD/DVD player, washing machine, fridge, electric fan, domestic phone, mobile phone, computer, internet access, camera/video camera, bicycle, motorcycle/scooter, three-wheeler, motor car/van, and bus/lorry/tipper.

Categorical variables were transformed into dichotomous indicators by creating separate dummy variables for each category. They consisted of the following (numbers in parentheses indicates number of categories in each): flooring material (5), material of wall (7), type of housing tenure (12), drinking water source (16), type of toilet (4), method of household garbage disposal (6), lighting power source (5), cooking fuel (13), and type of cooking place (3).

There was a small percentage of missing values in each variable (2–3%). These were imputed with either the PSU or stratum level mean of the variable or failing those the district/sector or national means. The principal component factor or index obtained by PCA after combining all these variables was then used to divide the sample into population weighted quantiles of equal size. Separate indices were not estimated for urban or rural sectors, but analysis indicates little difference between sectors in how the national index performs.
